# Efficacy and safety of vancomycin for local application in the prevention of surgical site infection after joint arthroplasty: a systematic review and meta-analysis

**DOI:** 10.1530/EOR-23-0023

**Published:** 2024-10-03

**Authors:** Chengxin Xie, Liwei Zhang, Dehua Zhang, Lingjian Tao, Yong Zhao, Hua Luo

**Affiliations:** 1 Department of Orthopedics, Taizhou Hospital of Zhejiang Province affiliated to Wenzhou Medical University, Taizhou, Zhejiang, China; 2 Department of Emergency, Taizhou Hospital of Zhejiang Province affiliated to Wenzhou Medical University, Taizhou, Zhejiang, China; 3 Department of Orthopedics, Shanghai Fengxian District Central Hospital, Shanghai 201400, China.

**Keywords:** arthroplasty, complication, infection, vancomycin

## Abstract

**Purpose:**

- The aim of this systematic review and meta-analysis is to explore the effect of topical vancomycin powder (VP) in surgical site infection (SSI) prevention and adverse events after joint arthroplasty and to provide a specific theoretical basis for clinical treatment.

**Methods:**

- The review process was conducted according to the PRISMA guidelines. Two independent researchers meticulously screened the literature based on predefined inclusion and exclusion criteria, evaluated the quality of the selected studies, and extracted relevant data. Data analysis was conducted using RevMan 5.4 software.

**Results:**

- This meta-analysis included 24 studies encompassing a total of 34 811 patients. The pooled analysis showed that the topical administration of VP significantly reduced the incidence of SSI. Subgroup analyses by doses, type of joint (hip and knee), and type of surgery (primary and revision) confirmed that vancomycin consistently lowered SSI rates. Moreover, the incidence of SSI caused by gram-negative germs and gram-positive germs decreased following the use of VP, although the reduction was not significant for infections caused by MRSA. However, the use of VP was associated with a significant increase in sterile complications at the incision site and delayed incision healing.

**Conclusion:**

- The topical application of VP is effective in reducing the incidence of infections following joint arthroplasty. Despite an increased risk of complications such as delayed healing of incisions, the pros and cons should be weighed in clinical decision-making. However, it should not be discarded due to side effects.

## Introduction

Joint arthroplasty is an effective treatment for end-stage osteoarthritis. It can correct joint deformities, reduce joint pain, restore joint function, and improve patients’ quality of life. However, several factors can affect the survival and efficacy of the knee replacement prosthesis, including poor alignment of the lower extremities, infection, aseptic loosening of the prosthesis, and anterior knee pain (1). Among these, surgical site infection (SSI) is the most common, with the incidence rate ranging from 0.69% to 2.2% (2, 3). The diagnosis of SSI is complex, and its treatment is challenging. When SSI occurs, it significantly impacts joint function, prolongs hospital stays, and increases treatment costs. In the United States, the average cost per person for joint infection is $93 600 (4), with the total cost of SSI-related joint infections estimated to reach $1.85 billion by 2030 (5). The systemic effects of topical vancomycin powder (VP) in the incision are minimal, allowing for a high local drug concentration that effectively kills bacteria. This efficacy has been demonstrated in spinal surgery (6). In 2009, Chiu *et al.* first used vancomycin with cement for knee revision patients and found that it could effectively reduce the risk of SSI (7).

Assor *et al.* found that topical administration of VP effectively reduced the incidence of SSI in patients undergoing initial non-cemented joint replacement (8). Subsequent large-scale retrospective studies have supported these findings (9, 10). However, other studies have reported that the topical application of VP did not significantly reduce the incidence of SSI compared to control groups (11, 12, 13). Given the relatively low incidence of SSI, clinical research in this area is often ambiguous and hampered by the small sample size. This makes it challenging to obtain high-quality clinical data, such as that derived from randomized controlled trials, in the arthroplasty literature. Several systematic reviews and meta-analyses have examined the use of local VP in joint arthroplasty, but their results have been inconsistent. Therefore, this study aims to systematically review the literature to determine the efficacy and complications associated with the topical application of VP in joint arthroplasty. We hypothesize that topical VP will reduce the incidence of SSI compared to the control group.

## Materials and methods

According to the PRISMA (Preferred Reporting Items for Systematic Reviews and Meta-Analyses) statement, this meta-analysis was performed in agreement (14). The protocol for this meta-analysis was registered on PROSPERO (Registration no. CRD 42022356822).

### Inclusion criteria

Study type: randomized controlled trial or cohort study, or retrospective study (level I to III evidence). Study population: patients undergoing joint arthroplasty. Intervention and control: topical VP in the treatment group, no VP in the control group. Outcome index: SSI or wound complication reported.

### Exclusion criteria

Letters, case reports, reviews, animal trials, or republished studies; unused vancomycin in the treatment group; previous joint infections; studies lacking a control group.

### Outcomes

The primary outcome was the incidence of SSI. Secondary outcomes were the incidence of complications.

### Search strategy

Two of the authors performed the search in PubMed, EMBASE, Ovid, Web of Science, CNKI, and the Cochrane Central Register of Controlled Trials from the inception dates to 09 December 2022, using the keywords ‘Vancomycin and (THA OR THR OR Arthroplasty, Replacement, Hip OR TKA OR TKR OR Arthroplasty, Replacement, Knee OR ((hip OR knee) adj2 (replace* OR arthroplast* OR prosthe*))) and (infect* or wound complication or wound breakdown or erosion or wound dehiscence or prolonged wound or delayed wound)’. No language restrictions were applied during the search.

### Study selection

Two researchers individually screened the retrieved literature strictly against inclusion and exclusion criteria. Initially, titles and abstracts were reviewed to identify documents that potentially met the inclusion criteria. These documents were then read in full to confirm their eligibility. In cases of disagreement between the two researchers during the screening process, a senior researcher was consulted to make the final decision.

### Data collection process

Data on relevant outcome measures were extracted from the literature that met the inclusion criteria, including author, year, study design type, country, sample size, vancomycin dose, follow-up, and number of SSI by two researchers individually.

### Assessment of risk of bias and quality of evidence

Two researchers independently assessed the quality of all included trials based on Cochrane risk-of-bias criteria (15). The Newcastle–Ottawa scale (NOS) was used to evaluate the literature quality of the retrospective studies (16). We also examined the quality of evidence for outcomes using the GRADE (grading of recommendations assessment, development, and evaluation) approach (17).

### Data synthesis

The meta-analysis was performed using RevMan (version 5.4; The Cochrane Collaboration) software. The heterogeneity was assessed using the Q test and I^2^ value calculation. If the heterogeneity was not present (*P* > 0.1 and *I*^2^ < 50%), the data were combined with a fixed-effect model. If the heterogeneity was present (*P* < 0.1 or *I*^2^ > 50%), the random-effects model was used. The odds ratio (OR) and its associated 95% CI were used to assess outcomes, and a *P-*value less than 0.05 suggested that the difference was statistically significant.

### Subgroup analyses

We performed subgroup analyses for similar subsets of patients across trials.

### Sensitivity analyses

We performed a sensitivity analysis by excluding the largest trial, cluster-randomized or quasi-randomized trials, excluding trials with a high risk of bias, and using random-effects models.

### Result

**Figure 1:**
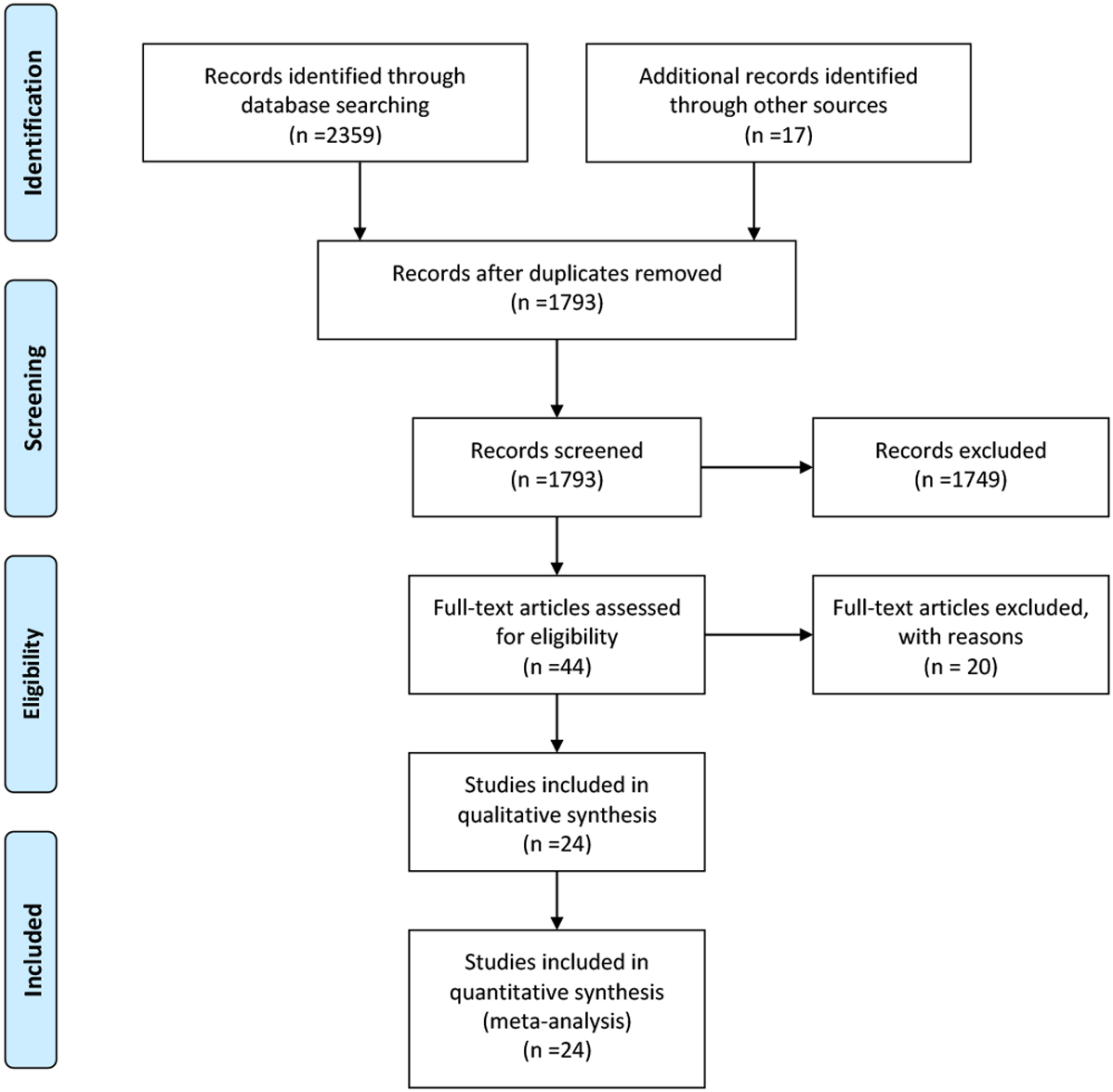
Flow diagram for search and selection of included studies.

According to our search strategy, a total of 2376 articles related to the application of VP in joint replacement were retrieved (PubMed: 300, Ovid: 1355, WoS: 514, Cochrane Library: 53, CNKI: 137, additional records identified through other sources: 17). After removing 583 duplicate articles, 1749 were excluded based on their titles and abstracts. Upon reading the full texts of the remaining 44 articles, 20 were excluded for the following reasons: two were conference reports, five did not discuss the topical use of vancomycin, four were unrelated to infection, two did not specify the number of infections or infection rates of SSIs, five were registered RCT studies without results, one focused on periprosthetic joint infection patients, and two were repeated reports from the same institution (18, 19). For these repeated reports, only the most recent study was included. (See Fig. 1 for a detailed flowchart). Ultimately, 24 articles with a total of 34 811 patients met the inclusion and exclusion criteria (7, 8, 9, 10, 11, 12, 13, 19, 20, 21, 22, 23, 24, 25, 26, 27, 28, 29, 30, 31, 32, 33, 34, 35). In Buchalter’s study (31), we extracted data from only two of the three groups, excluding the group with a high risk of infection. Although studies by Iorio *et al.* and Buchalter *et al.* originated from the same institution (26, 31), there was no overlap in the data extracted. The study from Iorio *et al.* included only high-risk patients, while the study from Buchalter *et al.* excluded the high-risk group, allowing us to include all cases from the study of Iorio *et al.* The study from Tahmasebi *et al.* mentioned cases of superficial infection but did not provide specific numbers, so it was not included in the overall infection rate calculation. Instead, its data were analyzed as a subgroup for periprosthetic joint infections (10). Detailed information on all included studies is presented in Tables 1 and 2. The quality of RCT studies was assessed using the Cochrane risk-of-bias tool, while retrospective studies were evaluated using the NOS score, with all included studies achieving high technical quality (≥6 stars) (Table 3).

**Table 1:** Characteristics of included studies.

Study	Country	Subjects, *n*	Study period	Age (mean ± s.d.)		Design	Participants	Duration
				VP	Control			
Aljuhani *et al.* (11)	Saudi Arabia	98	2018.01–2020.03	–	–	RS	Primary TKA	3 M
Assor *et al.* (8)	France	135	2002–2006	73 ± 8.2	72 ± 7.6	PS	Primary TKA	3–7 Y
Buchalter *et al.* (31)	USA	9228	2012.01–2013.12; 2016.01–2019.09	63.74 ± 9.54	63.82 ± 10.28	RS	Primary TKA	3 M
Chang *et al.* (35)	China	290	2014.06–2018.06	64.97 ± 7.66	71.08 ± 2.19	RS	Primary TKA	1 Y
Chiu *et al.* (7)	China	183	1993–2004	71 ± 8.4	70 ± 7.8	PS	Revision TKA	3 Y
Cohen *et al.* (12)	USA	555	2015.04–2016.12	66 ± 10.2	67.3 ± 12.6	RS	Primary cementless THA	–
Crawford *et al.* (30)	USA	1885	2011 and 2015	64.8 ± 10.2	63.3 ±11.9	RS	Primary THA	–
Dial *et al.* (29)	USA	265	2013.06–2016.02	61.2 ± 11.1	61.5 ± 10.5	RS	Primary THA	3 M
Erken *et al.* (28)	Turkey	93	2019.01–2019.12	81.88 ± 7.54	81.87 ± 6.57	RS	Partial hip replacement	–
Hanada *et al.* (27)	Japan	202	2010–2017	74.6 ± 8.4	73.3 ± 6.6	PS	Primary TKA and UKA	1 Y
Iorio *et al.* (26)	USA	4664	2009.01–2016.03	–	–	RS	Primary and revision (THA and TKA)	–
Khatri *et al.* (13)	India	115	2014.02–2016.01	–	–	RS	Primary TKA	6 M
Koutalos *et al.* (25)	Greece	290	2016.01–2017.02	67	68	PS	Primary THA and TKA	2 Y
Li *et al.* (19)	China	285	2016.10–2020.05	66.89 ± 2.42	66.30 ± 3.18	RS	Primary THA	3 M
Li *et al.* (34)	China	192	2018.06–2020.01	–	–	RS	Primary THA	1 Y
Matziolis *et al.* (9)	Germany	8945	2013–2018	69 ± 10	68 ± 9	RS	Primary THA and TKA	1 Y
Otte *et al.* (24)	USA	1640	2012.05–2014.04	66.0 ± 10.7	67.6 ± 11.0	RS	Primary and revision (hip and knee)	3 M
Patel *et al.* (23)	USA	460	2016.04–2017.10	63.6	64.9	RS	Primary THA and TKA	3 M
Tahmasebi *et al.* (10)	Iran	2024	2007.03–2018.12	64.99 ± 11.49	66.37 ± 8.9	RS	Primary TKA	1 Y
Tian *et al.* (33)	China	387	2010.01–2014.02	56.7 ± 11.1	53.1 ± 12.7	RS	Primary THA and TKA	2 Y
Winkler *et al.* (22)	USA	744	2012.01–2015.12	70	46	RS	Primary and revision (THA and TKA)	6 M
Xu *et al.* (21)	China	855	2015.05–2017.10	66.9 ± 9.9	67.1 ± 9.3	RS	Primary TKA	18 M
Yang *et al.* (32)	China	300	2011.06–2017.03	58.21	58.41	RS	Primary THA	6 M
Yavuz *et al.* (20)	Turkey	976	2012–2016	65.5 ± 10.7	63.4 ± 12.1	RS	Primary TKA	2 Y

M, months; PS, prospective study; RS, retrospective study; THA, total hip arthroplasty; TKA, total knee arthroplasty; Y, years.

**Table 2:** Intervention and outcomes reported in the included studies.

Study	Intervention		Outcomes	Definition of infection
	VP	Control		
Aljuhani *et al.* (11)	2 g VP	No VP	SSI; DI	–
Assor *et al.* (8)	1–2 g VP	No VP	SFI, DI, IKS score	Joint fluid culture
Buchalter *et al.* (31)	2 g VP + 0.35% PI	No VP and PI	PJI	CDC’s National Healthcare Safety Network criteria
Chang *et al.* (35)	1 g VP	No VP	SFI, DI, WC	Meeting on PJI definition
Chiu *et al.* (7)	1 g VP	No VP	SFI,DI, HSS knee score	Laboratory tests + joint fluid culture
Cohen *et al.* (12)	1 g VP	No VP	PJI	MSIS criteria
Crawford *et al.* (30)	1 g VP	No VP	Overall infection, DI	–
Dial *et al.* (29)	1 g VP	No VP	SFI, DI, sterile WC, acute renal failure	Meeting on PJI definition
Erken *et al.* (28)	1 g VP	No VP	SSI	–
Hanada *et al.* (27)	1 g VP	No VP	PFI, WC	Meeting on PJI definition
Iorio *et al.* (26)	2 g VP + PI	No VP and PI	PJI	CDC’s definitions
Khatri *et al.* (13)	1 g VP	No VP	Overall infection, SFI, DI	SFI: observed by wound inspection; DI: evaluated by wound exploration
Koutalos *et al.* (25)	2 g VP + I-A TXA	I-ATXA	Overall infection, SFI, DI	SFI: WHO criteria; DI: MSIS criteria
Li *et al.* (19)	1 g VP	No VP	SFI, PJI, WC	Meeting on PJI definition
Li *et al.* (34)	1 g VP	No VP	SFI, PJI, WC	–
Matziolis *et al.* (9)	1 g VP	No VP	PJI	MSIS criteria
Otte *et al.* (24)	1 g VP	No VP	PJI	MSIS criteria
Patel *et al.* (23)	1 g VP	No VP	Overall infection rate, PJI, SFI, acute renal failure	–
Tahmasebi *et al.* (10)	1 g VP	No VP	Suspected superficial incisional infection, PJI	Philadelphia consensus
Tian *et al.* (33)	1 g VP	No VP	SFI, DI	IDSA guideline
Winkler *et al.* (22)	2 g VP	No VP	PJI	–
Xu *et al.* (21)	0.5 g VP	No VP	SFI, PJI, WC	Joint bacterial culture
Yang *et al.* (32)	1 g VP	No VP	SFI, DI, WC	Meeting on PJI definition
Yavuz *et al.* (20)	2 g VP	No VP	PJI	Meeting on PJI definition

CDC, Centers for Disease Control and prevention; DI, deep infection; I-A, intra-articular; IDSA, Infectious Diseases Society of America; IKS, International Knee Society; MSIS, Musculoskeletal Infection Society; PI, povidone iodine lavage solution; PJI, periprosthetic joint infection; SFI, superficial infection; SSI, surgical site infection; TXA, tranexamic acid; VP, vancomycin powder; WC, wound complications.

**Table 3:** Newcastle–Ottawa Scale ratings. Each asterisk represents one point.

Study	Selection	Comparability	Exposure/outcome
Aljuhani *et al.* (11)	****	*	***
Assor *et al.* (8)	****	**	***
Buchalter *et al.* (31)	****	**	**
Chang *et al.* (35)	****	**	***
Chiu *et al.* (7)	****	**	***
Cohen *et al.* (12)	****	*	**
Crawford *et al.* (30)	****	*	**
Dial *et al.* (29)	****	**	***
Erken *et al.* (28)	****	–	***
Hanada *et al.* (27)	****	*	***
Iorio *et al.* (26)	****	–-	**
Khatri *et al.* (13)	****	–	***
Koutalos *et al.* (25)	****	**	***
Li *et al.* (19)	****	**	***
Li *et al.* (34)	****	**	***
Matziolis *et al.* (9)	****	**	***
Otte *et al.* (24)	****	*	***
Patel *et al.* (23)	****	**	***
Tahmasebi *et al.* (10)	****	**	***
Tian *et al.* (33)	****	**	***
Winkler *et al.* (22)	****	*	***
Xu *et al.* (21)	****	**	***
Yang *et al.* (32)	****	**	***
Yavuz *et al.* (20)	****	**	***

### Effectiveness of topical use of VP in SSI

**Figure 2:**
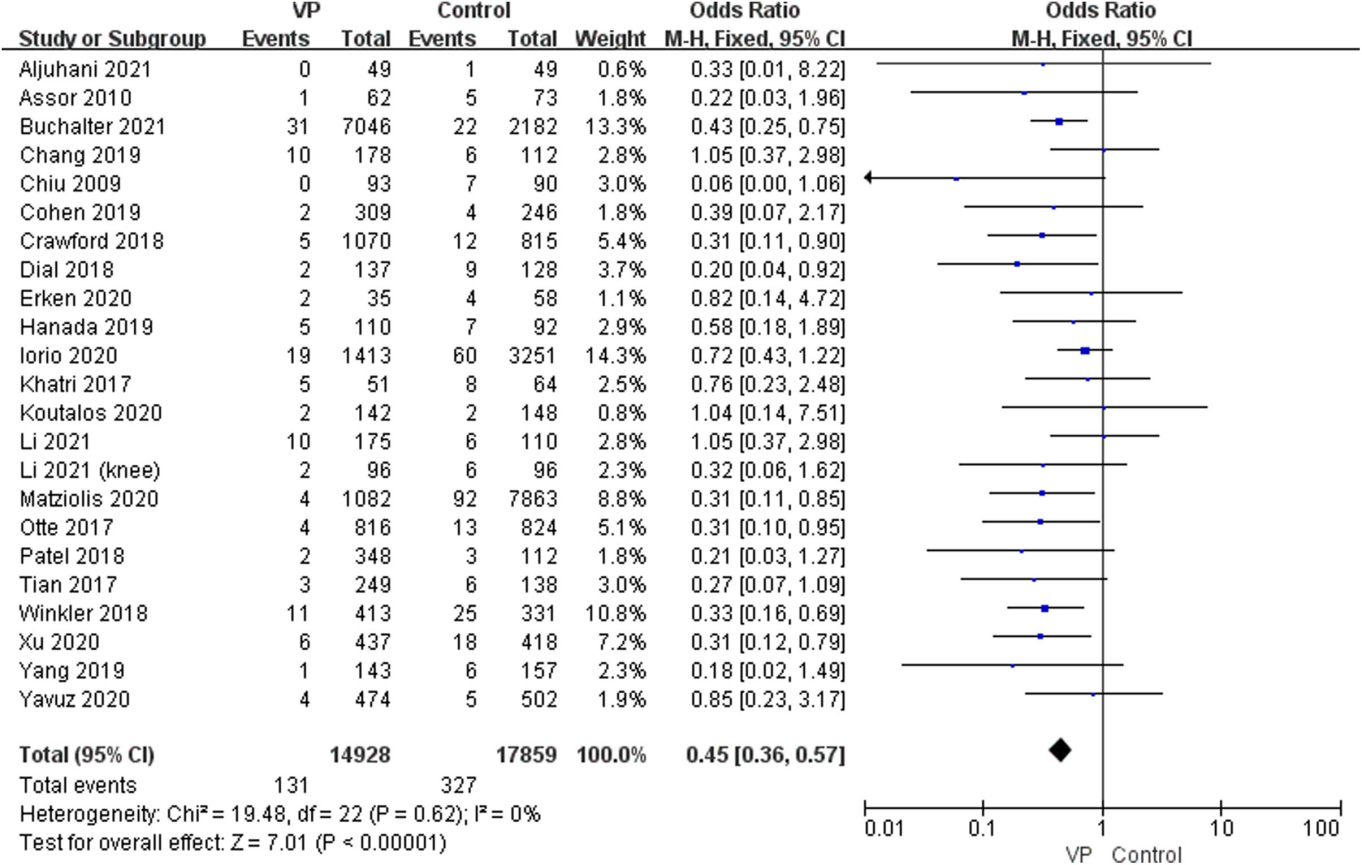
Forest plot of comparison: VP versus No VP; outcome: incidence of SSI (surgical site infection) (7, 8, 9, 11, 12, 13, 19, 20, 21, 22, 23, 24, 25, 26, 27, 28, 29, 30, 31, 32, 33, 34, 35).

A total of 23 studies reported on SSI (7, 8, 9, 11, 12, 13, 19, 20, 21, 22, 23, 24, 25, 26, 27, 28, 29, 30, 31, 32, 33, 34, 35). In the study by Iorio *et al.*, due to missing original data, there was an error in the calculation process. To address this, we used a rounding method to obtain the result and verified that adding or subtracting one unit did not affect the outcome. By combining the OR of 23 studies, we found no heterogeneity (*P* = 0.62, *I* ^2^ = 0%), and thus, the extracted data were merged using a fixed-effects model. The results showed that the infection rate in the intervention group was 0.88% (131/14 928) compared to 1.83% in the control group (327/17 859). The topical application of VP was effective in reducing the occurrence of SSI (OR: 0.45; 95% CI: 0.36–0.57, *P* < 0.00001, *I* ^2^ = 0%, moderate GRADE, Fig. 2 and Table 4).

**Table 4:** Grading of recommendations assessment, developing, and evaluation (GRADE) used to assess the systematic review outcomes.

Outcomes	Anticipated absolute effects^*^ (95% CI)		Relative effect, OR (95% CI)	Number of		GRADE evidence^‡^
	Risk with CON	Risk with VP		Subjects	Studies^†^	
SSI	18 per 1000	8 per 1000 (7–11)	0.45 (0.36–0.57)	32 787	23	⨁⨁⨁◯ : Moderate
SFI	16 per 1000	9 per 1000 (6–15)	0.57 (0.35–0.93)	5642	13	⨁⨁◯◯ : Low
PJI	16 per 1000	7 per 1000 (5–9)	0.42 (0.33–0.54)	34 718	23	⨁⨁⨁◯ : Moderate
1 g	17 per 1000	7 per 1000 (5–10)	0.41 (0.30–0.57)	15 797	15	⨁⨁⨁◯ : Moderate
2 g	18 per 1000	10 per 1000 (7–13)	0.54 (0.39–0.74)	16 000	6	⨁⨁◯◯ : Low
0.5–2g	47 per 1000	14 per 1000 (6–33)	0.29 (0.12–0.69)	990	2	⨁⨁⨁◯ : Moderate
Hip	16 per 1000	8 per 1000 (6–12)	0.51 (0.35–0.74)	12 181	13	⨁⨁◯◯ : Low
Knee	20 per 1000	9 per 1000 (7–11)	0.43 (0.32–0.56)	20 606	17	⨁⨁⨁◯ : Moderate
Primary	17 per 1000	8 per 1000 (6–10)	0.45 (0.35–0.57)	27 455	21	⨁⨁⨁◯ : Moderate
Revision	74 per 1000	13 per 1000 (5–35)	0.17 (0.06–0.45)	668	3	⨁⨁⨁⨁ : High
Inf. bacteria	21 per 1000	6 per 1000 (4–10)	0.29 (0.18–0.48)	6318	12	⨁⨁⨁◯ : Moderate
MRSA	1 per 1000	1 per 1000 (0–3)	0.57 (0.15–2.20)	6203	11	⨁⨁◯◯ : Low
G−	5 per 1000	2 per 1000 (1–4)	0.33 (0.14–0.81)	6203	11	⨁⨁⨁◯ : Moderate
G+	16 per 1000	4 per 1000 (2–7)	0.25 (0.14–0.46)	6203	11	⨁⨁⨁◯ : Moderate
AWC	35 per 1000	53 per 1000 (37–75)	1.52 (1.04–2.21)	2975	6	⨁⨁◯◯ : Low
PWH	34 per 1000	64 per 1000 (45–92)	1.93 (1.31–2.85)	2414	7	⨁⨁◯◯ : Low

*The risk in the intervention group (and its 95% CI) is based on the assumed risk in the comparison group and the relative effect of the intervention (and its 95% CI); ^†^All were observational studies; ^‡^GRADE Working Group grades of evidence – High certainty: we are very confident that the true effect lies close to that of the estimate of the effect; Moderate certainty: we are moderately confident in the effect estimate: the true effect is likely to be close to the estimate of the effect, but there is a possibility that it is substantially different; Low certainty: our confidence in the effect estimate is limited: the true effect may be substantially different from the estimate of the effect; Very low certainty: we have very little confidence in the effect estimate: the true effect is likely to be substantially different from the estimate of effect.

AWC, aseptic wound complications; CON, control; G−, Gram negative; G+, Gram positive; Inf., infectious; MRSA, methicillin-resistant Staphylococcus aureus; OR, odds ratio; PJI, periprosthetic joint infection; PWH, prolonged wound healing; SFI, superficial infection; SSI, surgical site infection.

### Subgroup analysis

**Figure 3:**
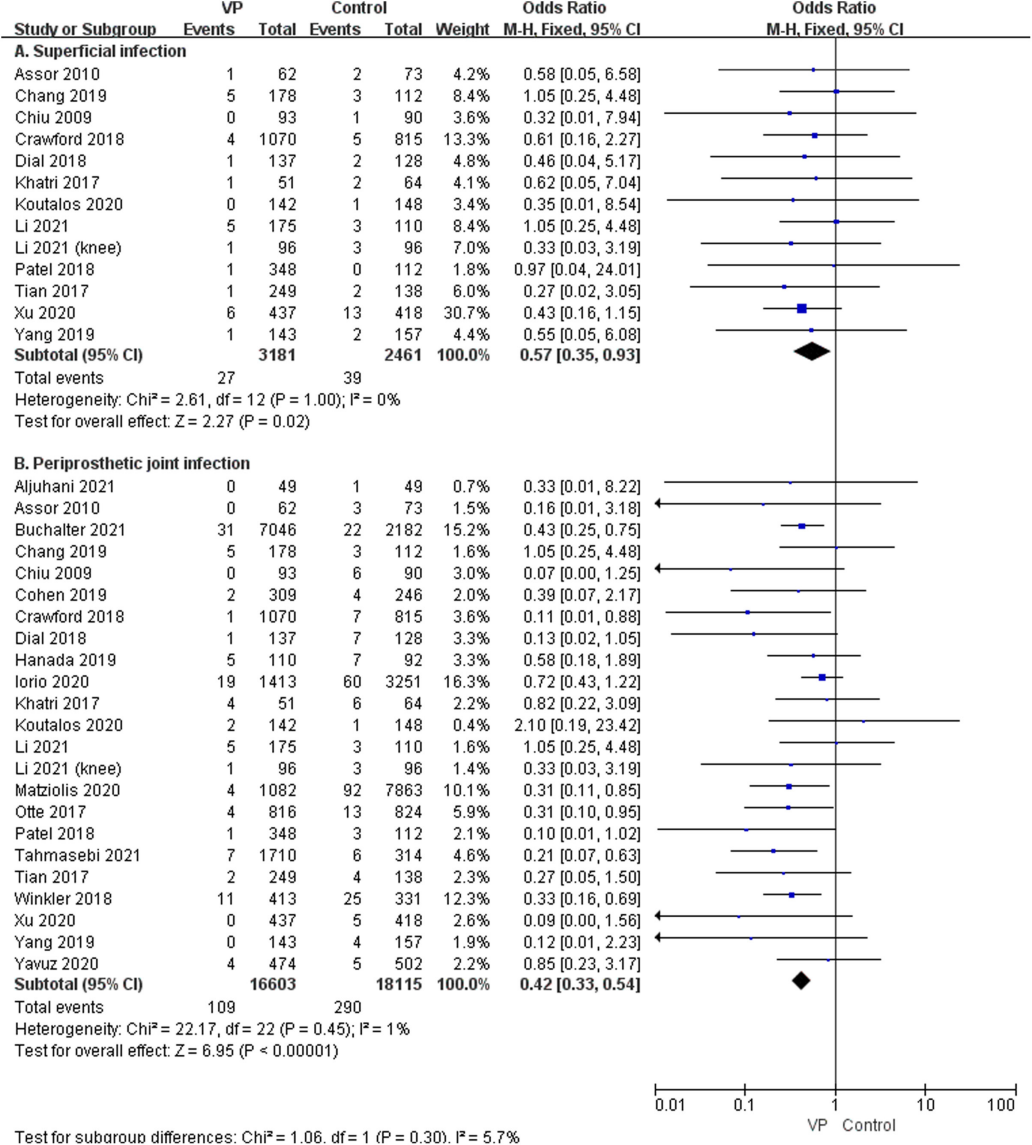
The subgroup analysis of the SSI (surgical site infection) in superficial (A) (7, 8, 13, 19, 21, 23, 25, 29, 32, 33, 34, 35, 36) and periprosthetic joint infection (B) (7, 8, 9, 10, 11, 12, 13, 19, 20, 21, 22, 23, 24, 25, 26, 27, 29, 31, 32, 33, 34, 35, 36).

**Figure 4:**
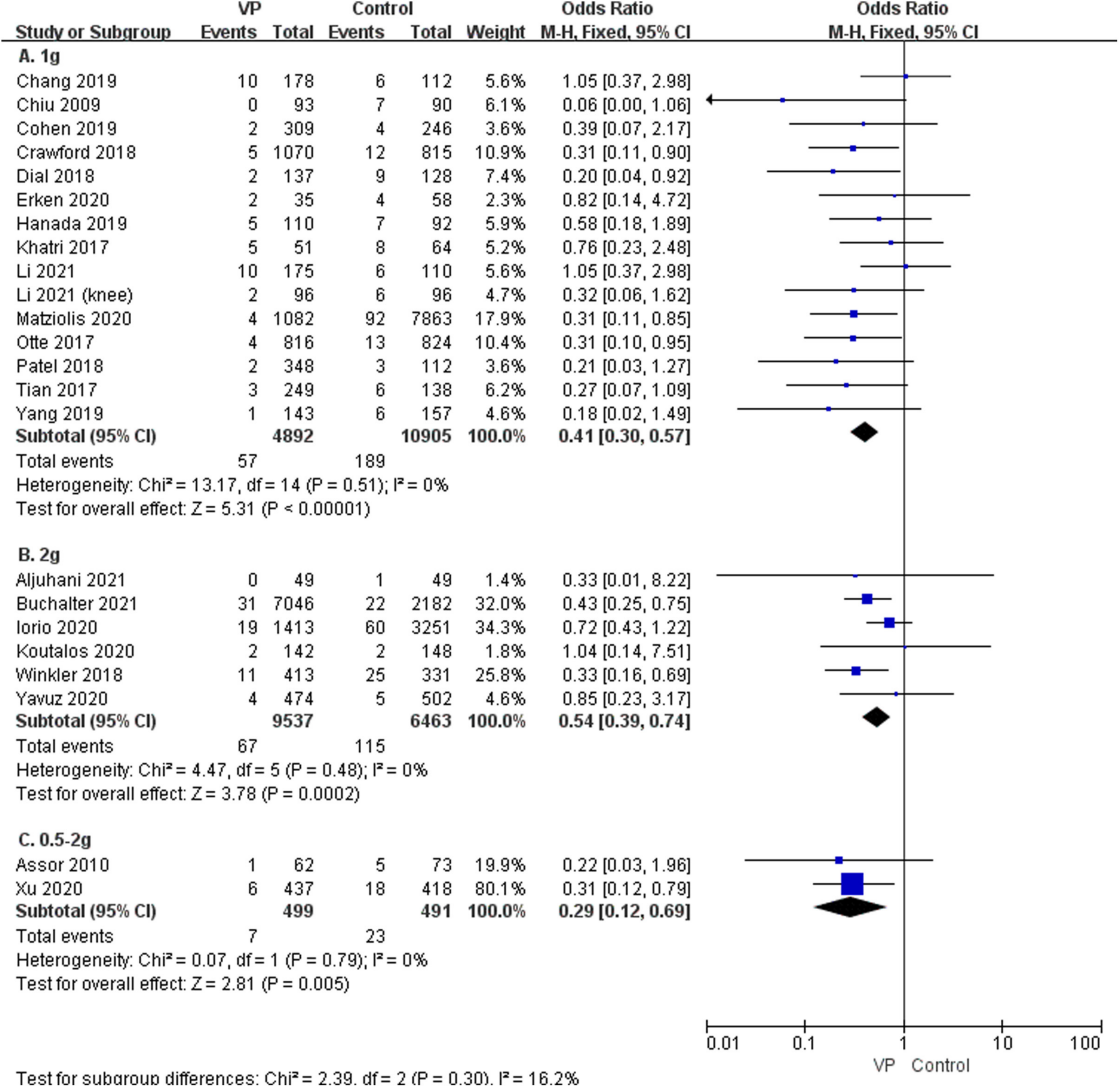
The subgroup analysis of the dosage of vancomycin powder used (A): 1g (7, 9, 12, 13, 19, 23, 24, 27, 28, 29, 32, 33, 34, 35, 36), (B): 2g (11, 20, 22, 25, 26, 31), (C): 0.5–2g (8, 21)).

**Figure 5:**
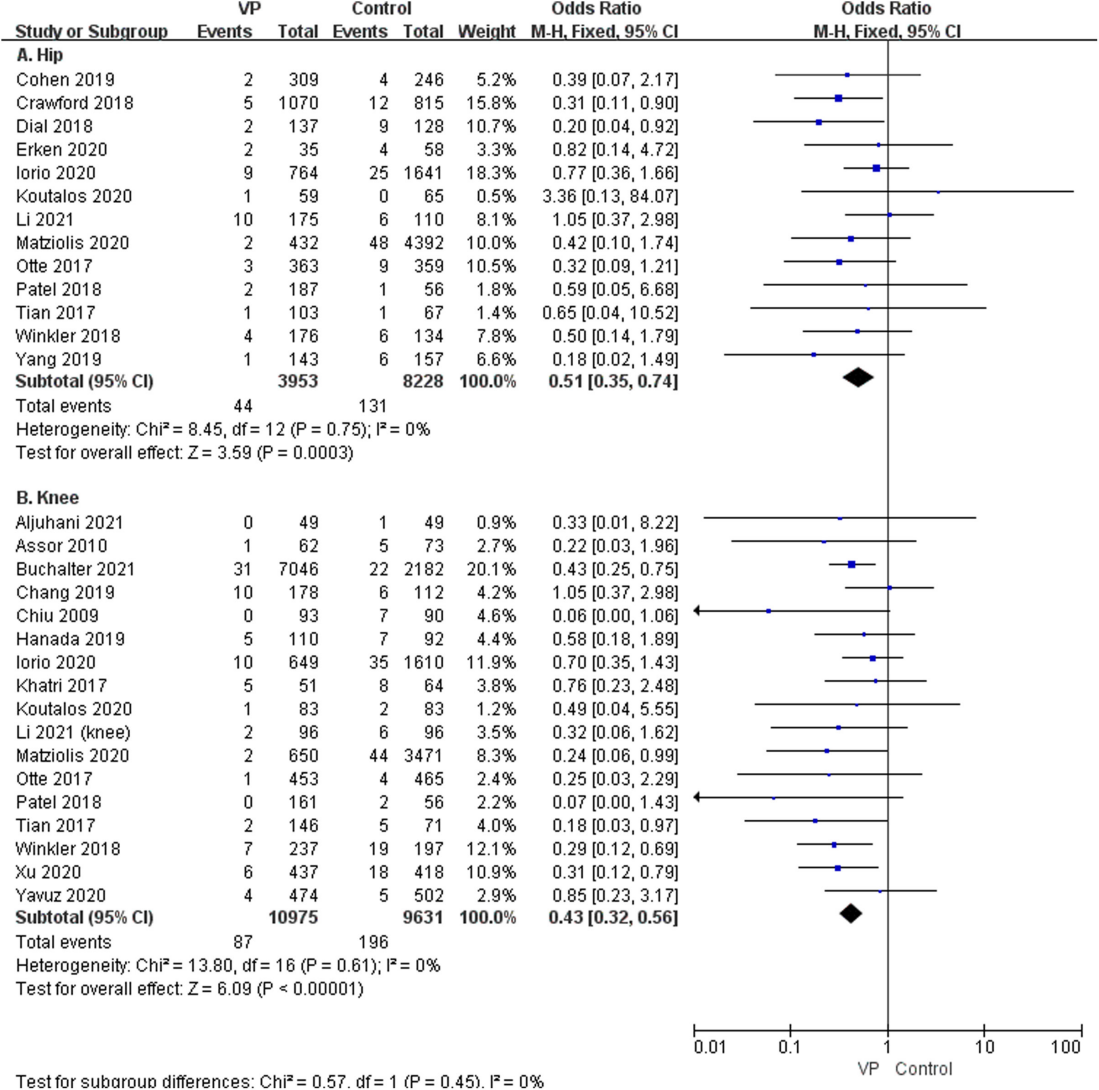
The subgroup analysis of the use of vancomycin powder in different surgical areas (A): Hip (9, 12, 19, 22, 23, 24, 25, 26, 28, 29, 32, 33, 36), (B): Knee (7, 8, 9, 11, 13, 20, 21, 22, 23, 24, 25, 26, 27, 31, 33, 34, 35)).

**Figure 6:**
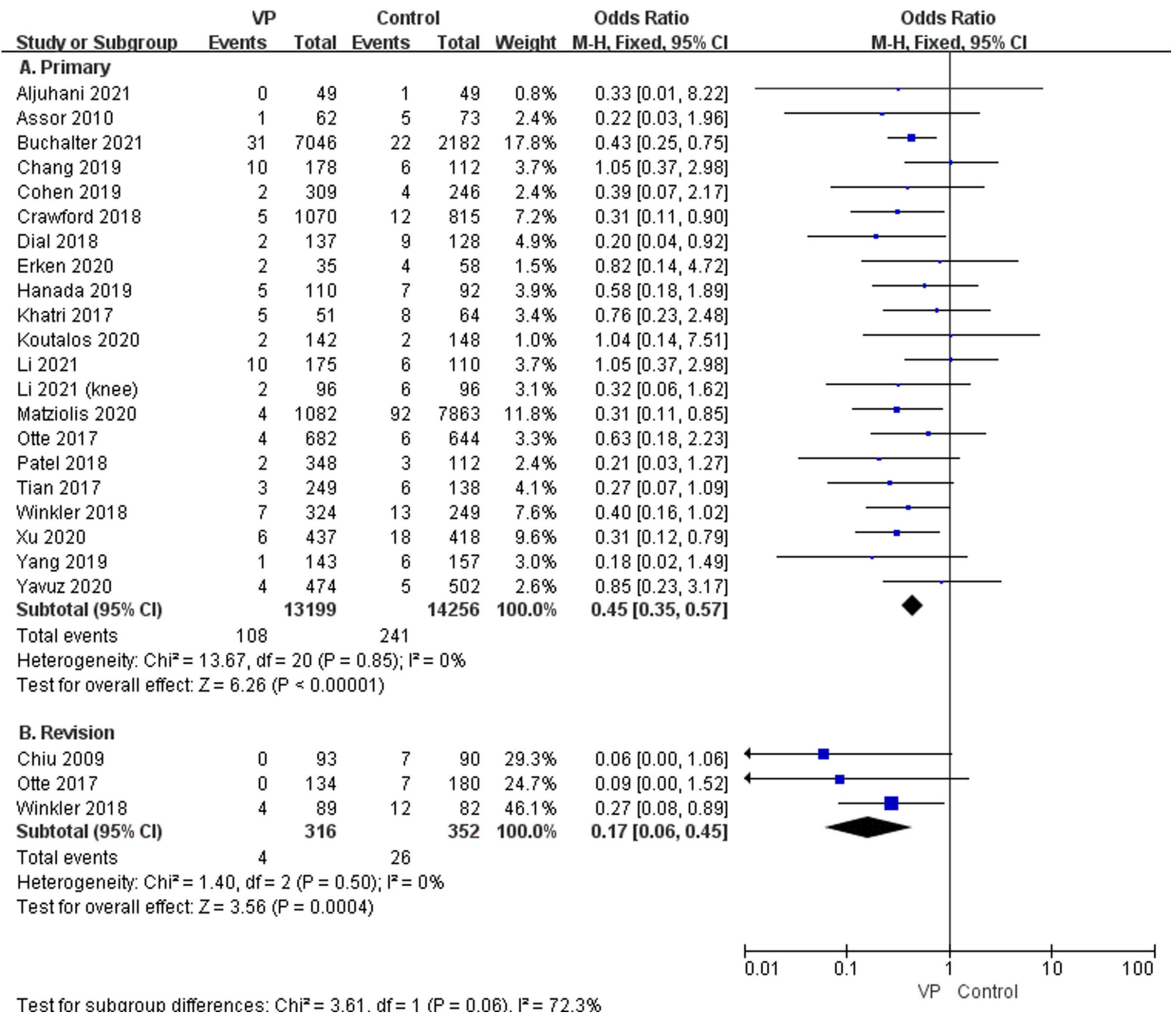
The subgroup analysis of the use of vancomycin powder in primary (A) (8, 9, 11, 12, 13, 19, 20, 21, 22, 23, 24, 25, 27, 28, 29, 31, 32, 33, 34, 35, 36) and revision (B) (7, 22, 24) arthroplasty.

Considering the use of different doses of vancomycin, varying surgical sites, primary versus revision surgery, and superficial versus periprosthetic joint infections, outcomes may vary. Therefore, a subgroup analysis was performed. The subgroup analysis demonstrated that the local application of VP had a significant preventive effect on both superficial infections (OR: 0.57, 95% CI: 0.35–0.93; *P* = 0.02, *I* ^2^ = 0%, low GRADE, Fig. 3A and Table 4) and periprosthetic joint infection (OR: 0.42, 95% CI: 0.33–0.54; *P* < 0.00001, *I*^2^ = 1%, moderate GRADE, Fig. 3B and Table 4). The result of the subgroup analysis on the dose of 1g, 2g, and 0.5–2g VP revealed that VP sprayed on the wound, at a dose of 1g (OR: 0.41, 95% CI: 0.30–0.57; *P* < 0.00001, *I*^2^ = 0%, moderate GRADE, Fig. 4A and Table 4), 2g (OR: 0.54, 95% CI: 0.39–0.74, *P* = 0.0002, *I*^2^ = 0%, low GRADE, Fig. 4B and Table 4), and 0.5–2g (OR: 0.29, 95% CI: 0.12–0.69; *P* = 0.005, *I*^2^ = 0%, moderate GRADE, Fig. 4C and Table 4), respectively, could reduce the occurrence of SSI after joint arthroplasty. We performed a subgroup analysis based on the surgical sites (hip vs knee). The results showed that VP could significantly decrease the SSI on hips (OR: 0.51, 95% CI: 0.35–0.74, *P* = 0.0003, *I*^2^ = 0%, low GRADE, Fig. 5A and Table 4) and knees (OR: 0.43, 95% CI: 0.32–0.56, *P* < 0.00001, *I*^2^ = 0%, moderate GRADE, Fig. 5B and Table 4). Furthermore, a subgroup analysis based on the type of surgery (primary or revision) showed that VP powder reduced the infection rate for both primary (OR: 0.45, 95% CI: 0.35–0.57, *P* < 0.00001, *I*^2^ = 0%, moderate GRADE, Fig. 6A and Table 4) and revision (OR: 0.17, 95% CI: 0.16–0.45, *P* < 0.00001, *I*^2^ = 0%, high GRADE, Fig. 6B and Table 4).

### The effect of local application of vancomycin on infectious bacteria

Twelve trials reported on the infectious bacteria (7, 8, 12, 13, 20, 21, 23, 27, 29, 32, 33, 36). When multiple bacteria were detected in the same patient, we categorized them into gram-negative or gram-positive bacteria. Each category recorded counts up to one case per patient, although the same patient might have multiple gram-negative or gram-positive bacteria. In Khatri’s study, multiple microbial infections were reported without specifying microbial names, so only the total number of bacteria detected was calculated in our meta-analysis (13). While Matsiolois *et al.* also reported specific cultured bacterial types (9), they classified all cultured bacteria collectively rather than by patient, leading to a higher number of events in our study than the true value. Consequently, this study was not included in the subgroup analysis. It is important to note that the number of pathogens may not equal the number of failures, as some patients had polymicrobial infections.

**Figure 7:**
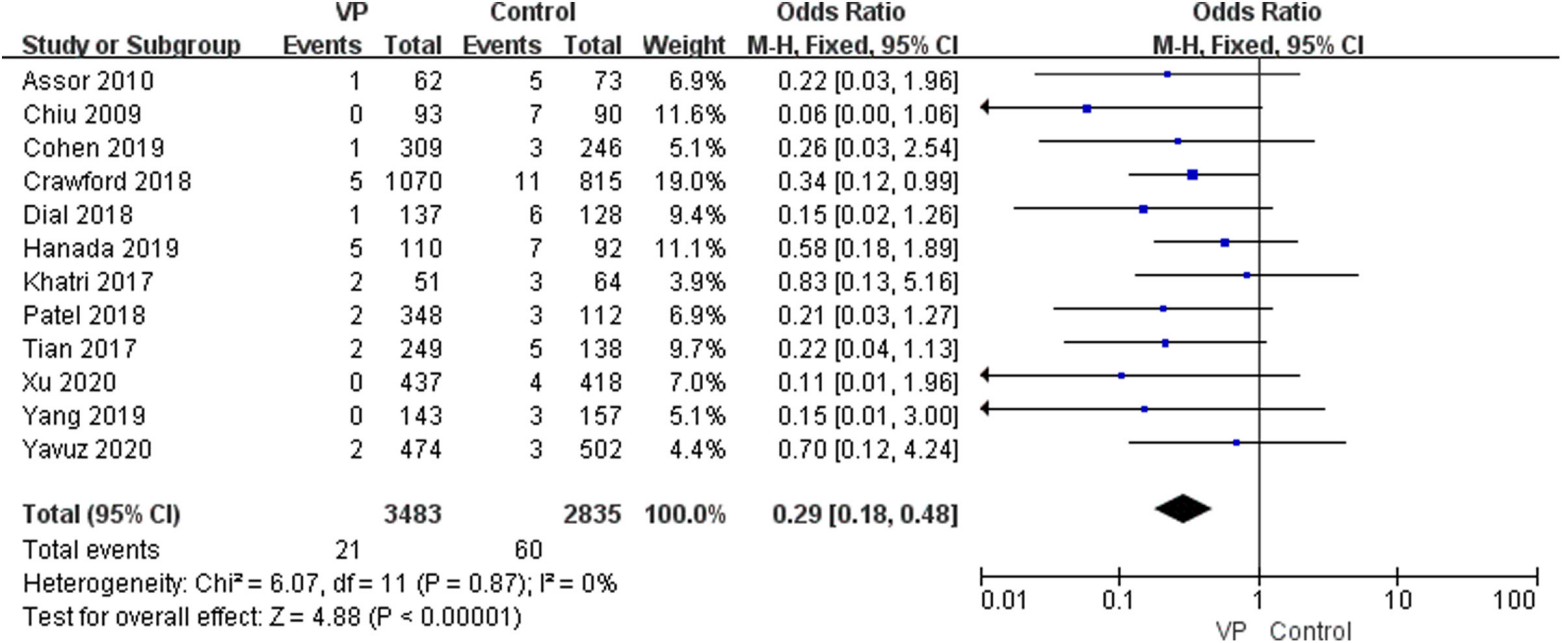
The effect of the local application of vancomycin on infectious bacteria (7, 8, 12, 13, 20, 21, 23, 27, 29, 32, 33, 36).

**Figure 8:**
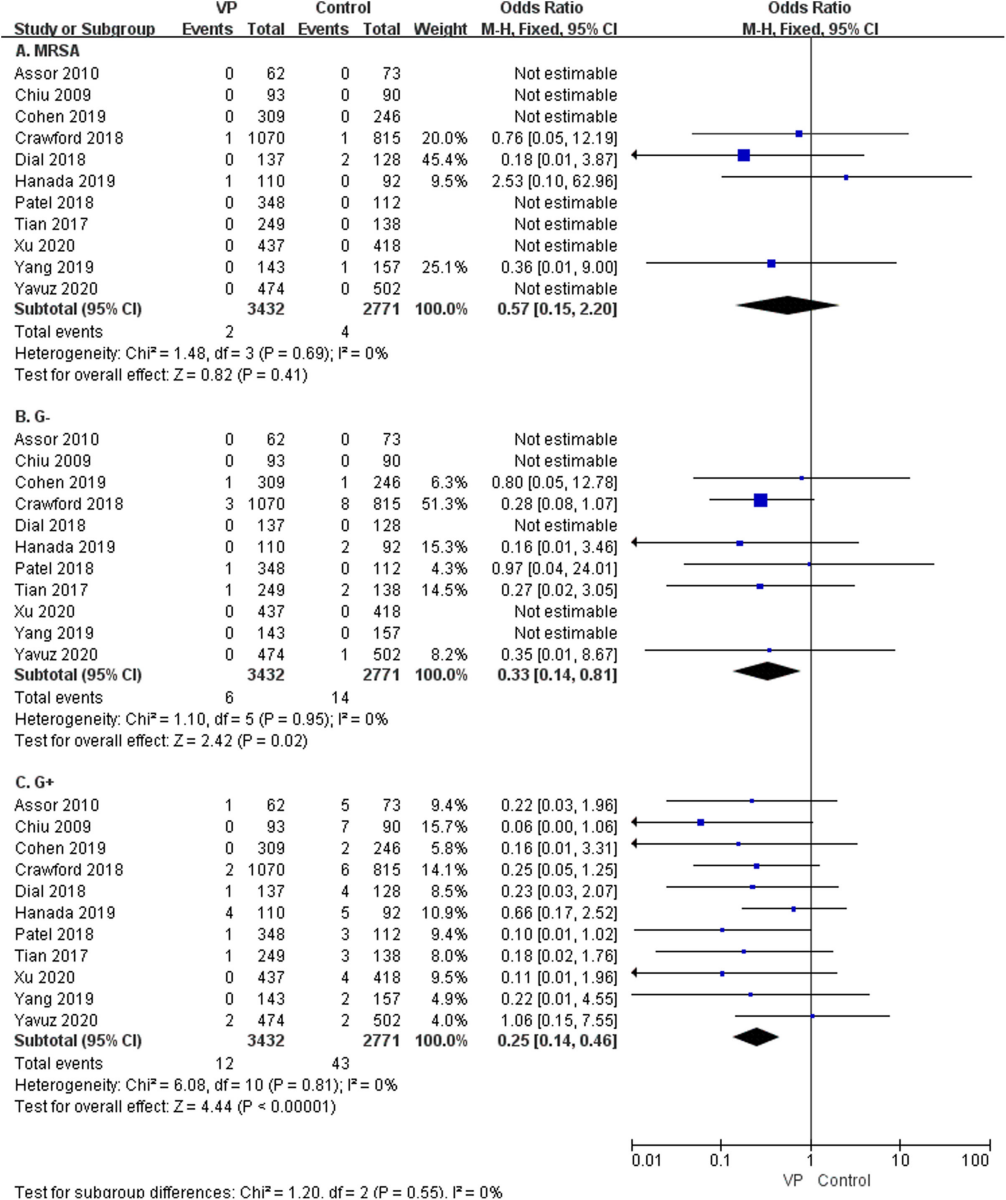
The subgroup analysis of different bacterial types (A): MRSA (7, 8, 12, 20, 21, 23, 27, 29, 32, 33, 36), (B): gram-negative bacteria (7, 8, 12, 20, 21, 23, 27, 29, 32, 33, 36), (C): gram-positive bacteria (7, 8, 12, 20, 21, 23, 27, 29, 32, 33, 36)).

The results showed that using vancomycin significantly reduces the positive rate of bacterial culture (OR: 0.29, 95% CI: 0.18–0.48, *P* < 0.00001, *I*^2^ = 0%, moderate GRADE, Fig. 7 and Table 4). A pooled subgroup analysis of different bacterial types revealed that vancomycin significantly reduces infection from gram-negative bacteria (OR: 0.33, 95% CI: 0.14–0.81, *P* < 0.02, *I*^2^ = 0%, moderate GRADE, Fig. 8B and Table 4) and gram-positive bacterial infections (OR: 0.25, 95% CI: 0.14–0.46, *P* < 0.00001, *I*^2^ = 0%, moderate GRADE, Fig. 8C and Table 4). However, it was not statistically significant in preventing MRSA infections (OR: 0.57, 95% CI: 0.15–2.20, *P* = 0.41, *I*^2^ = 0%, low GRADE, Fig. 8A and Table 4).

## Other adverse events

**Figure 9:**
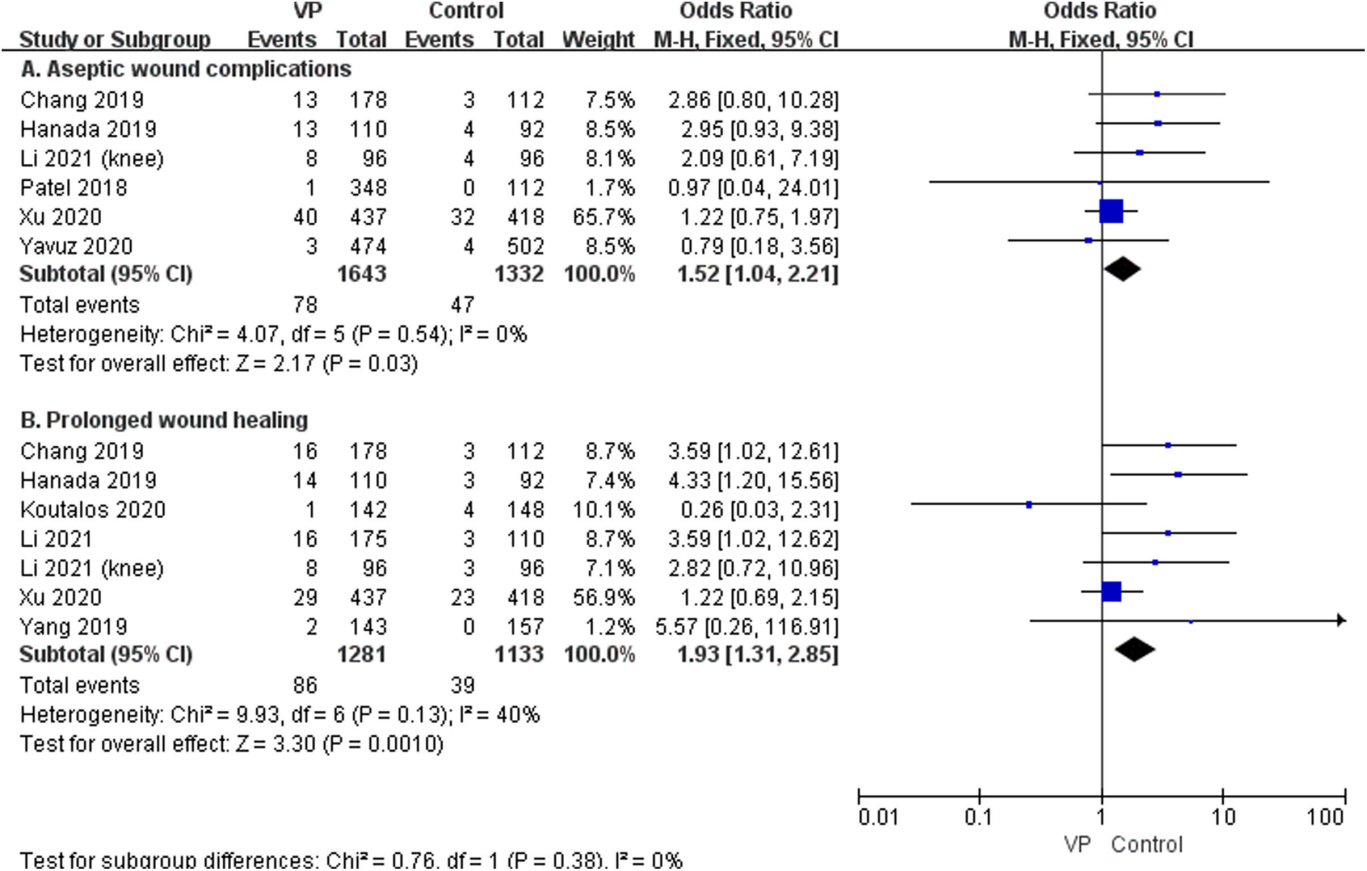
The effect of the local application of vancomycin on other adverse events (A): aseptic wound complications (20, 21, 23, 27, 34, 35), (B): prolonged wound healing (19, 21, 25, 27, 32, 34, 35)).

The pooled analysis showed that the use of VP increased the incidence rate of aseptic wound complications (OR: 1.52, 95% CI: 1.04–2.21, *P* = 0.03, *I*^2^ = 0%, low GRADE, Fig. 9A and Table 4) and prolonged wound healing (OR: 1.93, 95% CI: 1.31–2.85, *P* = 0.001, *I*^2^ = 40%, low GRADE, Fig. 9B and Table 4).

### Sensitivity analysis

A sensitivity analysis of the included studies was performed on a case-by-case exclusion basis. If any one study was excluded, the remaining studies were combined using the OR values. No studies had a significant impact on the results.

### Risk of bias

**Figure 10:**
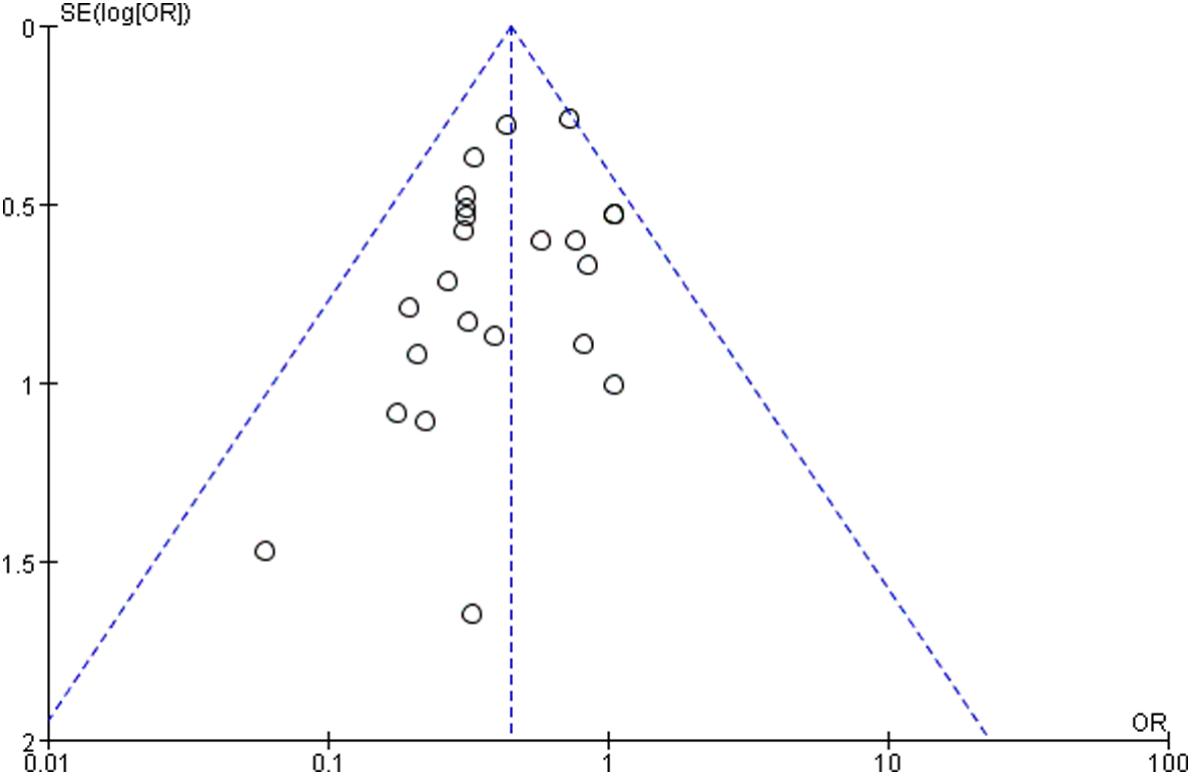
Funnel plot of the included studies in this meta-analysis for the incidence of surgical site infection.

Figure 10 shows that small sample studies may be the leading cause of bias.

## Discussion

Our meta-analysis included a total of 24 articles, all of which were retrospective or prospective studies. Despite the inherent limitations of these study designs, the NOS scores were greater than six points, providing some guiding significance for clinical practice. Our results align with previous meta-analyses, demonstrating that the topical application of vancomycin can effectively reduce the incidence of infection, whether in superficial or in periprosthetic joint infection. Analysis of different surgical sites, doses, and primary versus revision shows that the topical application of VP effectively reduces the risk of infection. Additionally, the use of VP can significantly lower the risk of local bacterial infections, especially those caused by G− and G+ bacteria. Although our findings indicate no difference in the MRSA detection after the topical application of vancomycin compared to the control group, this could be due to the high standard of sterile practices and environments currently in place, which already reduce the risk of MRSA infection. Further studies are needed to confirm this observation. Dial *et al.* reported a 4.4% incidence of complications in patients receiving VP (29). Due to the low pH of the vancomycin solution (2.8–4.5) (37), topical application of VP can lead to local skin necrosis. Our results showed that patients who received topical vancomycin had an increased incidence of aseptic wound complications compared to the control group. These complications may arise from a local inflammatory response to VP or from the body’s reaction to the external application of VP. While these incision complications are not as severe as SSIs, they still require additional treatment and, in some cases, secondary surgical intervention. This can lead to prolonged hospital stays, increased costs, and patient anxiety. Therefore, it is crucial to carefully consider these potential risks before applying VP.

Vancomycin is a glycopeptide antibiotic widely used to treat gram-positive bacterial infections. However, intravenous administration may not achieve sufficient minimum inhibitory concentrations (MIC) in local tissues (38, 39). Studies have shown that vancomycin-impregnated cement can achieve intraoperative bone concentrations up to 100 times the MIC, maintaining levels at four times the MIC even 6 months post-surgery (40). Studies have demonstrated that vancomycin concentrations below 2500 mg/L do not affect osteoblasts, while concentrations between 2500 and 5000 mg/L exhibit transient toxicity. Long-term exposure to 7500 mg/L can inhibit osteoblast function and cause cell death (41). Notably, the topical application of vancomycin involves much lower concentrations than these toxic levels, indicating that preventive doses are safe and non-toxic to surrounding bone and soft tissue cells. However, some researchers argue that suboptimal preventive effects may delay incision healing and increase the incidence of wound rupture due to secondary hematoma (27, 29). To this end, we conducted this meta-analysis study to evaluate the efficacy and complications associated with the topical application of vancomycin in joint arthroplasty.

Previous meta-analyses have examined whether topical vancomycin reduces infection rates after joint replacement (42, 43, 44, 45, 46, 47). However, these studies had limitations. Five meta-analyses included fewer than ten trials (43, 44, 45, 46, 47), and additional trials have since been published. Furthermore, some analyses focused solely on primary joint replacement, excluding revision arthroplasty. The meta-analysis performed by Movassaghi *et al.* included 16 trials (42), but had several limitations: First, only two databases were searched, and the language was limited to English. Secondly, the study focused on total joint arthroplasty, yet it included Hanada *et al.*’s trial on unicompartmental knee arthroplasty as total joint arthroplasty, introducing bias. Thirdly, in Koutalos *et al.*’s trial, it was unreasonable to merge data from groups not using vancomycin consistently; the experimental group combined tranexamic acid with vancomycin, whereas the control group should have used only tranexamic acid to control other variables. Fourthly, the analysis was limited to periprosthetic joint infections. Superficial infections, primarily caused by local skin bacteria, were not considered, despite their potential to lead to deep secondary infections if aggravated. Fifthly, three trials from the same institution were included and their data merged for analysis (26, 31, 48), increasing the risk of bias. Sixthly, Riesgo *et al.*’s study used a control group consisting of patients with periprosthetic joint infections, which may not align with the inclusion criteria for a standard meta-analysis.

Our study addresses these limitations by searching multiple databases without language restrictions. We conducted comprehensive subgroup analyses on vancomycin doses, superficial and periprosthetic joint infections, hip and knee surgeries, and primary versus revision arthroplasty. We also examined the effect of vancomycin on microbial detection and associated complications. During data processing, we ensured consistency in treatment and control groups, excluding duplicate cases from the same institution to minimize bias. This methodological rigor enhances the scientific validity of our findings.

### Limitation

Our article has the following limitations: First, different trials employed varying diagnostic criteria for infections, resulting in increased heterogeneity and potentially influencing the outcome indicators. Secondly, all included studies were retrospective, which generally provides a lower level of evidence. Thirdly, in some studies, patients allergic to penicillin received intravenous vancomycin for perioperative prophylaxis, introducing potential bias. Fourthly, while our findings suggest that vancomycin doses of 0.5–2 g can prevent infection, there is no standardized method or dosage for the topical application of vancomycin. Further research is needed to determine the optimal dosing regimen. Fifthly, the follow-up periods varied among the included trials, with two studies not specifying the follow-up duration. Follow-up periods shorter than 3 months may not capture all potential infections, leading to incomplete data. Sixthly, some experimental groups received additional treatments, such as dilute iodophor flushing and tranexamic acid, which could further reduce the incidence of events in the experimental group. Seventhly, SSI includes both superficial and periprosthetic joint infections. Several studies only reported outcomes for periprosthetic joint infections, without specifying superficial infections. By pooling SSI data using periprosthetic joint infection outcomes, we may have introduced bias into our results.

## Conclusion

Our study demonstrates that topical application of VP effectively reduces the occurrence of SSI, both superficial and periprosthetic joint infections. However, it also increases the incidence of aseptic wound complications and prolonged wound healing. Weighing the pros and cons, the use of VP on wounds to prevent SSI has some clinical value. Given the limitations of the currently included literature and the large sample size, further high-quality studies are needed to provide more reliable clinical evidence.

## ICMJE Conflict of Interest Statement

The authors declare that there is no conflict of interest that could be perceived as prejudicing the impartiality of the study reported.

## Funding Statement

This work did not receive any specific grant from any funding agency in the public, commercial, or not-for-profit sector.

## Author contribution statement

HL conceived the study. CX developed the research protocol. LT submitted the review to PROSPERO and performed the literature search. CX and DZ screened titles and abstracts and reviewed full texts. CX and LZ performed data abstraction. HL resolved discrepancies and reviewed the final dataset. HL performed data analyses and prepared the first manuscript draft. LZ and YZ contributed to the revision. All authors contributed to final edits and revisions prior to submission.
